# Hepatoprotection with a chloroform extract of *Launaea procumbens* against CCl_4_-induced injuries in rats

**DOI:** 10.1186/1472-6882-12-114

**Published:** 2012-08-03

**Authors:** Rahmat A Khan, Muhammad R Khan, Mushtaq Ahmed, Sumaira Sahreen, Naseer A Shah, Mir Sadiq Shah, Jasia Bokhari, Umbreen Rashid, Bushra Ahmad, Shumaila Jan

**Affiliations:** 1 Department of Biotechnology, Faculty of Biological Sciences, University of Science and Technology Bannu KPK, Bannu, Pakistan; 2 Department of Biochemistry, Faculty of Biological Sciences, Quaid-i-Azam University Islamabad, Islamabad, Pakistan; 3 Botanical Sciences Division, Pakistan Museum of Natural History, Garden Avenue, Shakarparian, Islamabad, Pakistan

**Keywords:** *Launaea procumbens*, Hepatic injuries, Flavonoids, Antioxidant enzymes, Carbon tetrachloride

## Abstract

**Background:**

*Launaea procumbens* (Asteraceae) is used as a folk medicine to treat hepatic disorders in Pakistan. The effect of a chloroform extract of *Launaea procumbens* (LPCE) was evaluated against carbon-tetrachloride (CCl_4_)-induced liver damage in rats.

**Methods:**

To evaluate the hepatoprotective effects of LPCE, 36 male Sprague–Dawley rats were equally divided into six groups. Animals of group 1 (control) had free access to food and water. Group II received 3 ml/kg of CCl_4_ (30% in olive oil v/v) via the intraperitoneal route twice a week for 4 weeks. Group III received 1 ml of silymarin *via* gavage (100 mg/kg b.w.) after 48 h of CCl_4_ treatment whereas groups IV and V were given 1 ml of LPCE (100 and 200 mg/kg b.w., respectively) after 48 h of CCl_4_ treatment. Group VI received 1 ml of LPCE (200 mg/kg b.w.) twice a week for 4 weeks. The activities of the antioxidant enzymes catalase, peroxidase (POD), superoxide dismutase (SOD), glutathione peroxidase (GSH-Px), glutathione S-transferase (GST), glutathione reductase (GSR), glutathione (GSH) and lipid peroxidation (thiobarbituric acid reactive substances (TBARS)) were measured in liver homogenates. DNA damage, argyrophilic nucleolar organizer regions (AgNORs) counts and histopathology were studied in liver samples. Serum was analyzed for various biochemical parameters. Phytochemical composition in LPCE was determined through high-performance liquid chromatography (HPLC).

**Results:**

LPCE inhibited lipid peroxidation, and reduced the activities of aspartate transaminase, alanine transaminase, alkaline phosphatase, and lactate dehydrogenase in serum induced by CCl_4_. GSH contents were increased as were the activities of antioxidant enzymes (catalase, SOD, GST, GSR, GSH-Px) when altered due to CCl_4_ hepatotoxicity. Similarly, absolute liver weight, relative liver weight and the number of hepatic lesions were reduced with co-administration of LPCE. Phyochemical analyses of LPCE indicated that it contained catechin, kaempferol, rutin, hyperoside and myricetin.

**Conclusion:**

These results indicated that *Launaea procumbens* efficiently protected against the hepatotoxicity induced by CCl_4_ in rats, possibly through the antioxidant effects of flavonoids present in LPCE.

## Background

The liver takes part in the metabolism, detoxification and secretion functions in the body. It is the major target organ of chemical-induced toxicity. Liver damage in most cases involves oxidative stress and is characterized by progressive evolution from steatosis to chronic hepatitis, fibrosis, cirrhosis, and hepatocellular carcinoma. More than 50% of individuals in the United States suffer from liver disorders [1,2]. Although the precise mechanisms of the pathogenesis of liver cirrhosis are incompletely understood, the role of free radicals and lipid peroxides has garnered considerable attention [3]. It has been found that the metabolism of carbon tetrachloride (CCl_4_) involves production of the highly lethal trichloromethyl radical (^·^CCl_3_) and peroxy trichloromethyl (^·^OOCCl_3_) free radical through activation by drug-metabolizing enzymes located in the endoplasmic reticulum [4,5]. CCl_4_ can cause lipid peroxidation as well as deposition of the extracellular matrix (ECM), resulting in liver cirrhosis [6,7]. Clinical and experimental examinations have shown that cirrhosis can be reversed. Various pharmaceutical drugs have been used to minimize and reverse the insult, but most of them lead to appreciable side-effects during long-term treatment. In this context, the use of an effective alternative without side-effects is crucial to reduce the oxidative stress which leads to hepatic disorders [8,9]. Currently, there is great awareness of the health benefits of phenolic and polyphenolic compounds because of their antioxidant potential [10-12]. Dietary plants possessing phenolic and polyphenolic compounds have been shown to exert various biological actions. These include the scavenging of free radicals, metal chelation, increases in enzymatic activity. More recently, they have been shown to influence signal transduction, release of transcription factors, and gene expression. They have received considerable attention in the past decade because of their reputed role in the prevention of several human disorders [13].

Medicinal plants possess quantities of phenolic and polyphenolic constituents, and are also in demand for use as functional foods or biopharmaceutical products. *Launaea procumbens* (LP) is an important medicinal plant used extensively in Ayurvedic and herbal medicine in Pakistan (which promotes self-remedy, good health and longevity) as well as being used as a food ingredient [14]. Traditionally, it has been used in the treatment of kidney disorders, hormonal imbalance and sexual diseases [15]. According to Shaukat et al. [16] the ethanolic extracts of LP have been used against pathogenic fungi. Chemical characterization has revealed that LP is composed of salicylic acid, vanillic acid, synergic acid, 2-methyl-resorcinol and gallic acid [16] as well as phenolic and polyphenolic compounds [17]. These compounds have spasmogenic, cardiovascular, anti-carcinogenic, anti-inflammatory, hepatoprotective and antioxidant properties [18]. The present study was designed to screen the chloroform fraction of LP for phytochemical composition using high-performance liquid chromatography (HPLC) as well as to evaluate its hepatoprotective potential against CCl_4_-induced hepatotoxicity.

## Methods

### Ethical approval of the study protocol

The study protocol was approved by an Ethics Committee of Quaid-i-Azam University for the Feeding and Care of Laboratory Animals.

### Plant collection

LP was collected from Wah Cantonment in the district of Rawalpindi (Pakistan) at maturity during June 2006. It was identified and a specimen submitted at the Herbarium of Pakistan (Quaid-i-Azam University, Islamabad, Pakistan). Aerial parts of the plant (leaves, stem, flowers, seeds) were dried in the shade at room temperature for 2 weeks. They were then chopped and ground mechanically to a mesh size of 1 mm.

### Preparation of plant extract

A total of 1.5 kg powder of LP was extracted with 2 L of absolute methanol in a separating funnel with refluxing for 5 h. The extract was cooled at room temperature, filtered, and evaporated under reduced pressure in a rotary evaporator. It was suspended in distilled water and fractionated with *n*-hexane, ethyl acetate and chloroform. The chloroform fraction of *Launaea procumbens* (LPCE) was dried and stored at 4°C for *in-vivo* studies.

### HPLC of LPCE

A total of 500 mg of LPCE was extracted with 6 ml of 25% hydrochloric acid and 20 ml of chloroform for 1 h. The obtained extract was filtered to a volumetric flask. The residue was heated twice with 20 ml of chloroform for 20 min to obtain the extract. The combined extract was diluted with chloroform to 100 ml. A 5-ml portion of the solution was filtered and transferred to a volumetric flask and diluted with 10 ml of chloroform. The sample (10 μl) was injected into the HPLC apparatus. Samples were analyzed on an Agilent HPLC system (Agilent, Santa Clara, CA, USA). Separation was carried out through a 20RBAX Eclipse XDB-C18 column (5 μm; 4.6 × 150 mm, Agilent) with a ultraviolet–visible (UV–vis) Spectra-Focus detector with an autosampler. Solvent A (0.05% trifluoroacetic acid) and solvent B (0.038% trifluoroacetic acid in 83% acetonitrile (*v/v*) were employed with the following gradient: 0–5 min, 15% B in A, 5–10 min, 50% B in A, 10–15 min, 70% B in A. The flow rate was 1 ml/min and the injection volume was 10 μl. Six standard compounds (myricetin, catechin, vitexin, kaempferol, hyperoside, rutin) were run for comparative detection and optimized. Calibration curves were defined for each compound in the range of sample quantity 0.02–0.5 μg. All samples were assayed in triplicate. All quantitative data were evaluated using analytic software.

### Animals

Thirty-six male Sprague–Dawley ratss (age, 6 weeks; 190–200 g) were provided by the National Institute of Health (Islamabad, Pakistan). They were kept in standard cages at room temperature (25 ± 3°C) with a 12-h dark–light cycle. They were allowed to consume standard laboratory food and water.

### Experimental design

To study the antioxidant possessions of LP, rats were equally divided into six groups. Group 1 (control) have free access to food. Group II received 3 ml/kg of CCl_4_ (30% in olive oil) via the intraperitoneal route twice a week for 4 weeks. Group III received silymarin 100 mg/kg body weight (b.w.) via the oral route after 48 h of CCl_4_ treatment. Groups IV and V were given 100 mg/kg b.w. and 200 mg/kg b.w. LPCE, respectively, after 48 h of CCl_4_ treatment as described above, while group VI received only LPCE at 200 mg/kg b.w. for 4 weeks. Twenty-four hours after the last treatment, all rats were weighed and their blood collected; they were then killed. Livers were removed, weighed, and perfused in ice-cold physiological (0.9%) saline solution. Half of the liver was reated with liquid nitrogen for enzymatic and DNA-damage analyses, whereas the other portion was processed for histological analyses.

### Assessment of levels of liver marker enzymes and biochemical parameters

Serum analyses of various liver marker enzymes alanine transaminase (ALT), aspartate transaminase (AST), alkaline phosphatase (ALP), gamma-glutamyl transpeptidase (γ-GT), lactate dehydrogenase (LDH)), total cholesterol (TC), high-density lipoprotein- cholesterol (HDL-C), low-density lipoprotein-cholesterol (LDL-C) and triglycerides (TGs) were estimated using standard AMP Diagnostic Kits (Graz, Austria).

### Assessment of levels of antioxidant enzymes

Hepatic tissues were homogenized in 10 volumes of 100 mmol KH_2_PO_4_ buffer containing 1 mmol ethylenediamine tetra-acetic acid (EDTA; pH 7.4) and centrifuged at 12,000 × *g* for 30 min at 4°C. The supernatant was collected and used for the assessment of antioxidant enzymes. Protein concentrations in the supernatants of liver tissue homogenates were determined using crystalline bovine serum albumin (BSA) as a standard. All chemicals used in enzymatic analyses were purchased from Sigma–Aldrich (St Louis, MO, USA).

### Catalase assay

Catalase activity was determined using the method of Chance and Maehly [19] with some modifications. The reaction solution of catalase activity contained 2.5 ml of 50 mmol phosphate buffer (pH 5.0), 0.4 ml of 5.9 mmol H_2_O_2_ and 0.1 ml of hepatic supernatant. Changes in the absorbance of the reaction solution at 240 nm were determined after 1 min. One unit of catalase activity was defined as an absorbance change of 0.01 as units/min.

### Superoxide dismutase (SOD) assay

The SOD activity of liver tissue was estimated using the method of Kakkar et al. [20]. he reaction mixture contained 0.1 ml of phenazine methosulfate (186 μmol), 1.2 ml of sodium pyrophosphate buffer (0.052 mmol; pH 7.0), 0.3 ml of the supernatant after centrifugation (1500 × *g* for 10 min followed by 10,000 × *g* for 15 min). The enzyme reaction was initiated by adding 0.2 ml of NADH (780 μmol) and stopped after 1 min by the addition of 1 ml of glacial acetic acid. The amount of chromogen formed was measured by recording color intensity at 560 nm. Results are expressed in units/mg protein.

### Glutathione-S-transferase (GST) assay

GST activity was assayed using the method of Habig et al. [21]. The reaction mixture consisted of 1.475 ml of phosphate buffer (0.1 mol, pH 6.5), 0.2 ml of reduced glutathione (1 mmol), 0.025 ml of CDNB (1 mmol) and 0.3 ml of homogenate in a total volume of 2.0 ml. Changes in absorbance were recorded at 340 nm, and GST activity was calculated as nmol CDNB conjugate formed/min/mg protein using a molar extinction coefficient of 9.6 × 10^3^ M^–1^ cm^–1^.

### Glutathione Reductase (GSR) assay

GSR activity was determined using the method of Carlberg and Mannervik [22]. The reaction mixture consisted of 1.65 ml of phosphate buffer: (0.1 mol; pH 7.6), 0.1 ml of EDTA (0.5 mmol), 0.05 ml of oxidized glutathione (1 mmol), 0.1 ml of nicotinamide adenine dinucleotide phosphate (NADPH) (0.1 mmol) and 0.1 ml of homogenate in a total volume of 2 ml. Enzyme activity at 25°C was estimated by measuring the disappearance of NADPH at 340 nm. It was calculated as nmol NADPH oxidized/min/mg protein using a molar extinction coefficient of 6.22 × 10^3^ M^–1^ cm^–1^.

### Glutathione peroxidase (GSH-px) assay

GSH-Px activity was assayed using the method of Mohandas et al. [23]. The reaction mixture consisted of 1.49 ml of phosphate buffer (0.1 mol; pH 7.4), 0.1 ml of EDTA (1 mmol), 0.1 ml of sodium azide (1 mmol), 0.05 ml of GSR (1 IU/ml), 0.05 ml of reduced glutathione (GSH; 1 mmol), 0.1 ml of NADPH (0.2 mmol), 0.01 ml of H_2_O_2_ (0.25 mmol) and 0.1 ml of homogenate in a total volume of 2 ml. The disappearance of NADPH at 340 nm was recorded at 25°C. Enzyme activity was calculated as nmol NADPH oxidized/min/mg protein using a molar extinction coefficient of 6.22 × 10^3^ M^–1^ cm^–1^.

### Quinone Reductase (QR) assay

The activity of QR was determined using the method of Benson et al. [24]. The 3-ml reaction mixture consisted of 2.13 ml of Tris–HCl buffer (25 mmol; pH 7.4), 0.7 ml of BSA, 0.1 ml of FAD, 0.02 ml of NADPH (0.1 mmol) and 0.l ml of homogenate. The reduction of dichlorophenolindophenol (DCPIP) was recorded at 600 nm. Enzyme activity was calculated as nmol of DCPIP reduced/min/mg protein using a molar extinction coefficient of 2.1 × 10^4^ M^–1^ cm^–1^.

### GSH assay

GSH was estimated using the method of Jollow et al. [25]. A total of 1.0 ml of homogenate was precipitated with 1.0 ml of 4% sulfosalicylic acid. Samples were kept at 4°C for 1 h and then centrifuged at 1200 × *g* for 20 min at 4°C. The total volume of 3.0 ml assay mixture contained 0.1 ml of a filtered aliquot, 2.7 ml of phosphate buffer (0.1 mol; pH 7.4) and 0.2 ml of DTNB (100 mmol). The yellow color that developed was read immediately at 412 nm on a SmartSpec™ Plus Spectrophotometer (Bio-Rad, Hercules, CA, USA). It was expressed as μmol GSH/g tissue.

### Estimation of lipid peroxidation using levels of thiobarbituric acid reactive substances (TBARS)

The assay for lipid peroxidation was carried out using a modified method of Iqbal et al. [26]. One milliliter of 20% TCA aqueous solution and 1.0 ml of 0.67% TBA aqueous solution was added to 0.6 ml of phosphate buffer (0.1 M; pH 7.4) and 0.4 ml of homogenate sample. The reaction mixture was heated in a boiling water-bath for 20 min and then moved to a bath of crushed ice before centrifugation at 2500 × *g* for 10 min. The amount of TBARS formed in each of the samples was assessed by measuring the optical density of the supernatant at 535 nm using a spectrophotometer against a reagent blank. Results were expressed as nmol TBARS/min/mg tissue at 37°C using a molar extinction coefficient of 1.56 × 10^5^ M^–1^ cm^–1^.

### Nitrite assay

A nitrite assay was conducted using Griess reagent. Tissue was deproteinized using equal volumes of 0.3 mol NaOH and 5% ZnSO_4_, and centrifuged at 6400 × *g* for 20 min, and the supernatant collected. A total of 1.0 ml of Griess reagent was added into the cuvette, and the spectrophotometer blanked at 540 nm. Then 20 μl of supernatant was added in a cuvette containing Griess reagent. Nitrite concentration was calculated using a standard curve for sodium nitrite.

### DNA fragmentation assay

The DNA fragmentation assay was conducted using the procedure of Wu et al. [27] with some modifications. Tissue (50 mg) was homogenized in 10 volumes of a TE solution at pH 8.0 (5 mmol Tris–HCl, 20 mmol EDTA) and 0.2% Triton X-100. A 1.0-ml aliquot of each sample was centrifuged at 27,000 × *g* for 20 min to separate the intact chromatin (pellet, B) from the fragmented DNA (supernatant, T). Pellet and supernatant fractions were assayed for DNA content using a freshly prepared diphenylamine (DPA) solution for the reaction. The optical density was read at 620 nm using the SmartSpec Plus Spectrophotometer. Results are expressed as % fragmented DNA using the following formula:

(1) $$\%\;\text{Fragmented DNA}=\text{T}\times 100/\left(\text{T},+,\text{B}\right)$$

### DNA ladder assay

DNA was isolated using the methods of Wu et al. [27] to estimate DNA damage. A 5-μg aliquot of DNA of rats was separately loaded in 1.5% agarose gel containing 1.0 μg/ml of ethidium bromide, including DNA standards (0.5 μg per well). Electrophoresis was done for 45 min at 100 V. After electrophoresis, gels were studied under a Gel Doc system and photographed using a digital camera.

### Argyrophilic nucleolar organizer regions (AgNORs) counts

A silver staining technique was used according to the methods of Trere et al. [28]. Unstained fixed slides were dewaxed by dipping for 3 min in xylene and were hydrated in decreasing ethanol concentrations (90%, 70% and 50%). After drying, slides were treated with one drop of colloidal solution (2% gelatin and 1% formic acid) and two drops of 50% AgNO_3_ solution onto the slides and incubated at 35°C for ≈8–12 min. Progressive staining was followed under a light microscope (Dialux 20 EB, Leitz, Wetzlar, Germany) to obtain golden-colored nuclei and brown/black NORs. Then, slides were washed in distilled water, treated for 1 min with 1% sodium thiosulfate at room temperature to stop the reaction, and washed in tap water. Cells were examined under a light microscope at 100 × magnification and the number of AgNORs per cell counted.

### Histopathological studies

For microscopic evaluation, liver samples were fixed in a fixative (absolute alcohol 60%, formaldehyde 30%, glacial acetic acid 10%) and embedded in paraffin, sectioned at 4-μm thickness, and subsequently stained with hematoxylin and eosin (H&E). Sections were studied under a light microscope at 40 × magnification. Slides of all treated groups were photographed and studied.

### Statistical analyses

To determine the treatment effects, one-way analysis of variance was carried using SPSS 13.0 computer software (SPSS, Chicago, IL, USA). The level of significance among the various treatments was determined by least squares difference (LSD) analyses at 0.05% and 0.01% levels of probability.

## Results

### HPLC quantification of flavonoids

**Figure 1:**
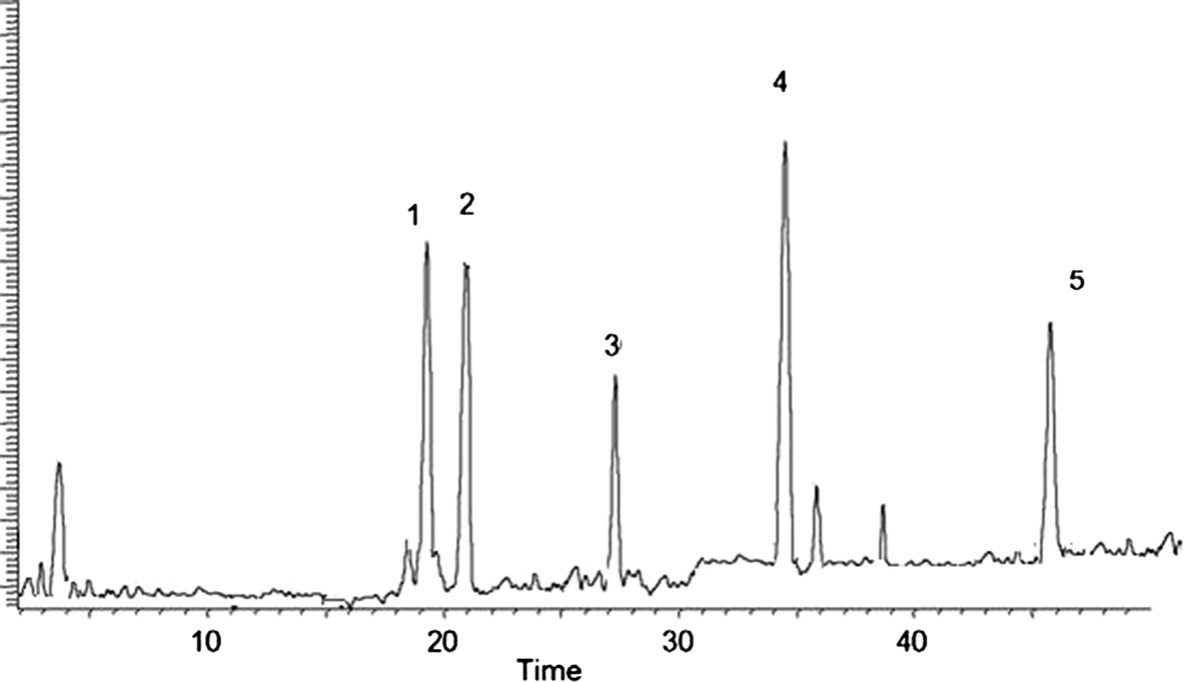
**HPLC fingerprints obtained by chloroform extracts of*L. procumbens*Column:C18 20RBAX ECLIPSE, XDB-C18, (5 μm; 4.6 × 150 mm, Agilent USA) eluted with mixtures trifluoroacetic acid and acetonitrile indicated the presence of five compounds kaempferol, catechin, myricetin, hyperoside and rutin.**

The investigated compounds in the LPCE were quantified by integration of the peak areas at 220 nm using an external calibration method for each analyte (Table 1). The main flavonoids in the extract were catechin, kaempferol, rutin, hyperoside and myricetin (Figure 1)along with some unidentified flavonoids (Table 2).

**Table 1:** Calibrations of standards

**Compound**	**Rt (min)**	**a**	**b**	**r**	**Linear range (ppm)**	**LOD (ppm)**
Kaempferol	19.2	625	-300	0.983	20-200	3.05
Catechin	21.0	12833	363.3	0.9419	10-170	2.30
Myricetin	27.2	12833	133.4	0.9792	4-125	1.60
Hyperoside	34.6	6333	153.3	0.989	6-165	0.85
Rutin	46.1	5250	110	0.9885	7-250	1.05

The relationship between peak area and analyte concentration is expressed as linear regression lines (*y* = *ax* + *b*), where *y* is the peak area measured by UV detector, *x* is the concentration (ppm) of the analytes, and *a* and *b* are the respective slope and intercept of the calibration curve. The correlation coefficient is *r.*

**Table 2:** HPLC quantification of various flavonoids of chloroform extract of *L. procumbens*

**Compound**	**Rt (min)**	**Quantity**	**Formula**	**Molecular weight**
Kaempferol	19.2	0.58±0.012	C_15_H_10_O_6_	286.23 g/mol
Catechin	21.0	0.97±0.072	C_15_H_14_O_6_	290.26 g/mol
Myricetin	27.2	0.47±0.035	C_15_H_10_O_8_	318.2351 g/mol
Hyperoside	34.6	0.77±0.003	C_21_H_20_O_12_	464.38 g/mol
Rutin	46.1	0.38±0.04	C_27_H_30_O_16_	610.517 g/mol

Mean ±SE (n=3 number).

### Effect of LPCE on body weight, liver w ight and AgNORs

There was a significant decrease (*P <* 0.01) in the body weight whereas absolute liver weight, relative liver weight and AgNORs count (NORs/cell) were increased significantly with CCl_4_ treatment as compared with the control group. There was a consistent increase in body weight whereas the absolute liver weight, relative liver weight and AgNORs count decreased with LPCE treatment. These parameters were also restored by silymarin treatment. However, LPCE alone did not induce a significant change (*P >* 0.05) in body weight, absolute liver weight, relative liver weight or AgNORs count in comparison with the control group (Table 3).

**Table 3:** Effects of LPCE on body weight, liver weight, relative weight, DNA damages and AgNORS

**Treatment**	**Liver weight (g)**	**Relative liver weight (% to body weight)**	**% Change in Body weight**	**AgNORS (NORS/cell)**	**DNA damages %**
Control	5.78±0.209++	0.0578± 0.00209++	26.0±0.80++	2.167±0.307++	5.17±0.94++
3 ml/kg CCl_4_	6.96±0.194**	0.0696±0.00194**	18.6±0.72**	9.000±0.931**	35.83±0.14**
100 mg/kg Silymarin+ CCl_4_	5.88±0.206++	0.0588± 0.00206++	25.9±0.63++	2.667±0.333++	5.00±0.44++
100 mg/kg LPCE+CCl_4_	5.98±0.128++	0.0598±0.00128++	23.2±0.71++	4.667±0.422++	16.0±0.55++
200 mg/kgLPCE+CCl_4_	5.81±0.218++	0.0531±0.00218++	25.3±0.47++	3.333±0.494++	5.17±0.17++
200 mg/kg LPCE alone	5.56±0.0760++	0.0526±0.00076++	25.9±0.42++	2.10±0.601++	4.90±0.67++

Mean ±SE (n=6 number).

** indicate significance from the control group at *P<0.01* probability level.

++ indicate significance from the CCl_4_ group at *P<0.01* probability level.

### Effect of LP on cholesterol profile

**Figure 2:**
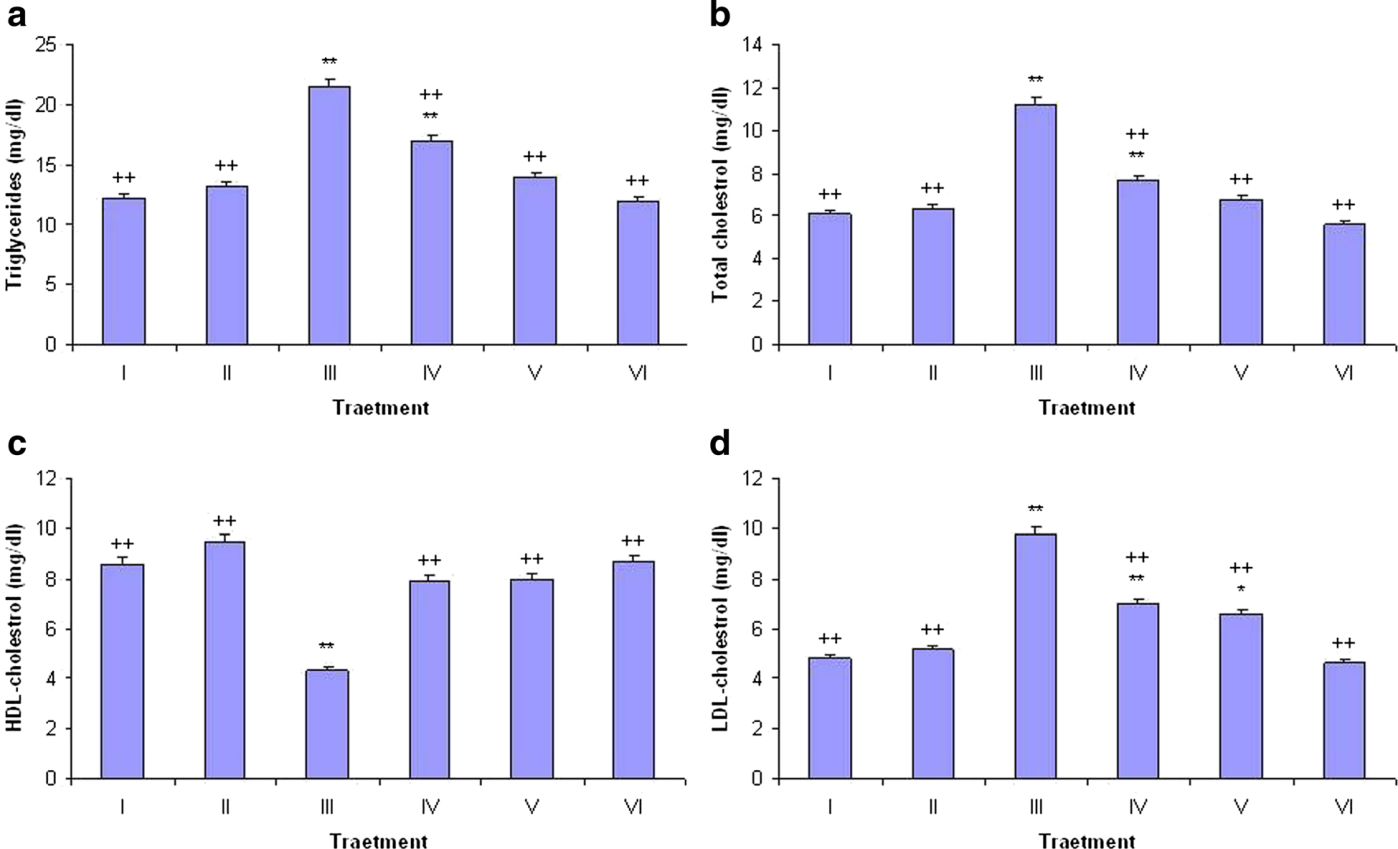
**Effect of*Launaea procumbens*on triglycerides (a), total cholesterol (b), HDL-cholesterol (c) and LDL-cholesterol (d); I (Control), II (CCl**_**4**_**), III (100 mg/kg Silymarin + CCl**_**4**_**), IV (100 mg/kg b.w., LPCE + CCl**_**4**_**), V (200 mg/kg b.w., LPCE + CCl**_**4**_**) and VI (200 mg/kg b.w., LPCE alone).**

The effect of LPCE on cholesterol profile is shown in Figure 2 a–d. Administration of CCl_4_ significantly (*P* < 0.01) increased the concentration of TGs, TC and LDL-C but decreased the HDL-C level as compared with the control group. Decrease in the HDL-C level was significantly (*P* < 0.01) restored with LPCE along with CCl_4_ treatment at both doses of LPCE (100 mg/kg b.w. and 200 mg/kg b.w.) whereas levels of TGs, TC and HDL-C were significantly (*P* < 0.01) increased only with LPCE at the administration of 200 mg/kg b.w. to offset the CCl_4_ insult. Silymarin significantly restored the cholesterol profile similar to that seen with the higher dose of LPCE. Treatment of LPCE alone to rats did not cause a significant alteration in the biochemical parameters stated above as compared with the control group.

### Indices of hepatotoxicity: liver marker enzymes

Administration of CCl_4_ markedly increased (*P* < 0.01) the activity of liver serum marker enzymes such as AST, ALT, ALP, LDH and γ-GT as compared with the control group (Table 4). Elevation in the secretion of these enzymes was significantly decreased (*P* < 0.01) by 200 mg/kg b.w. of LPCE and silymarin as compared with the CCl_4_ group. However, non-significant (*P* > 0.05) variation was observed by administration of 200 mg/kg b.w. of LPCE alone as compared with the control group.

**Table 4:** Effect of LPCE on liver marker enzymes

**Treatment**	**ALT (U/L)**	**AST (U/L)**	**ALP (U/L)**	**γ-GT (U/L)**	**LDH (nM/min/ mg protein)**
Control	32.17±2.12++	83.83±2.74++	248.00±3.93++	70.50±2.23++	48.3±2.38++
3 ml/kg CCl_4_	91.33±3.42**	228.00±4.27**	505.33±6.49**	119.33±3.12**	77.6±2.46**
100 mg/kg Silymarin+ CCl_4_	32.50±2.05++	84.67±2.75++	249.67±3.68++	71.33±2.04++	49±2.03++
100 mg/kg LPCE+CCl_4_	79.17±3.19*++	135.83±6.10**++	378.8±10.5**++	100.67±3.12**++	57±3.7**++
200 mg/kgLPCE+CCl_4_	40.17±2.09++	101.00±3.33++	260.33±3.61++	77.00±3.59++	50±2.96++
200 mg/kg LPCE alone	31.17±1.82++	82.33±3.77++	247.00±3.99++	71.17±2.41++	45±1.88++

Mean ±SE (n=6 number).

*, ** indicate significance from the control group at *P<0.05* and *P<0.01* probability level.

++ indicate significance from the CCl_4_ group at *P<0.01* probability level.

### Effect of the plant extract on parameters of oxidative stress in the liver

Antioxidant polyphenolic compounds have a key role in the detoxification of reactive oxygen species (ROS) and help to maintain cellular balance. Administration of CCl_4_ significantly decreased (*P* < 0.01) the activity of catalase, SOD, GST, GSH-Px, GSR and QR. Treatment of rats with 200 mg/kg b.w., of LPCE or silymarin (100 mg/kg b.w.) in combination with CCl_4_ significantly (*P* < 0.01) normalized the level of the antioxidant enzymes stated above. Non-significant changes (*P* > 0.05) were found by only feeding LPCE (Table 5).

**Table 5:** Effect of LPCE on hepatic oxidative stress parameters

**Treatment**	**CAT (U/min)**	**SOD (U/mg protein)**	**GST (nmol/ min/mg protein)**	**GSH-Px (nmol/ min/mg protein)**	**GSR (nmol/ min/mg** **protein)**	**QR (nmol/ min/mg protein)**
Control	4.397±0.275++	24.0±2.27++	128.50±4.62++	77.50±3.38++	147.33±6.01++	163.0±7.47++
3 ml/kg CCl_4_	2.590±0.240**	13.50±1.34**	68.83±4.57**	51.83±2.89**	88.00±3.61**	89.0±3.69**
100 mg/kg Silymarin+ CCl_4_	4.242±0.407++	23.0±2.34++	126.67±4.21++	77.0±3.10++	145.33±6.23++	160.0±6.97++
100 mg/kg LPCE+CCl_4_	3.407±0.276**++	18.83±1.49**++	100.83±5.47**++	69.0±3.4*++	112.1±4.1**++	116.6±4.3**++
200 mg/kgLPCE+CCl_4_	3.8567±0.07++	21.97±1.67++	123.83±1.96++	72.0±2.27++	135.17±3.39++	141.5±4.9++
200 mg/kg LPCE alone	4.40±0.105++	25.17±2.24++	132.67±4.45++	79.8±3.03++	149.33±6.27++	165.50±5.02++

Mean ±SE (n=6 number).

** indicate significance from the control group at *P<0.01* probability level.

++ indicate significance from the CCl_4_ group at *P<0.01* probability level.

### Effects of LPCE on levels of TBARS, GSH and nitrite

Free radicals combine with polyunsaturated fatty acids (PUFAs) to cause lipid peroxidation and increase TBARS contents in hepatic samples. The protective effects of LPCE on TBARS, GSH and nitrite levels among the hepatic samples of various groups of rats are shown in Table 6. Administration of CCl_4_ significantly reduced (*P* < 0.01) the concentration of GSH but increased (*P* < 0.01) TBARS contents and nitrite levels in hepatic samples as compared with the control group. Levels of TBARS and nitrite were significantly (*P* < 0.01) restored by the administration of 200 mg/kg b.w. of LPCE if administered with CCl_4_. Similarly, GSH contents were significantly (*P* < 0.01) increased by treatment with LPCE and silymarin as compared with the CCl_4_-treated group. However, non-significant (*P* > 0.05) changes were found with LPCE alone as compared with the control group.

**Table 6:** LPCE effects on TBARS, GSH and nitrite concentration of rat

**Treatment**	**GSH (mol/g tissue)**	**TBARS (nmol/min/mg protein)**	**Nitrite (mol/ml)**
Control	0.738±0.0201++	78.67±6.56++	56.33±2.46++
3 ml/kg CCl_4_	0.236±0.0066**	158.83±8.57**	79.0±3.88**
100 mg/kg Silymarin+ CCl_4_	0.708±0.0105++	79.00±7.45++	57.0±2.73++
100 mg/kg LPCE+CCl_4_	0.417±0.0135**++	108.67±4.29**++	60.67±1.48**++
200 mg/kgLPCE+CCl_4_	0.560±0.0181*++	91.33±6.77++	56.17±2.48++
200 mg/kg LPCE alone	0.743±0.0108++	77.83±3.86++	53.67±2.39++

Mean ±SE (n=6 number).

*, ** indicate significance from the control group at *P<0.05* and *P<0.01* probability level.

++ indicate significance from the CCl_4_ group at *P<0.01* probability level.

### Effect of LPCE on DNA damage

**Figure 3:**
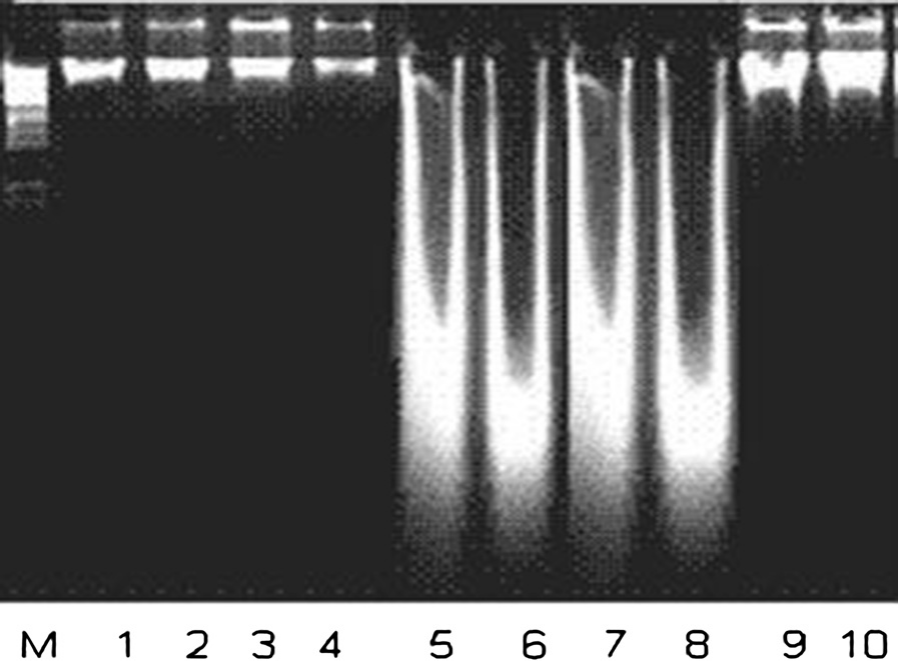
**Agarose gel showing DNA damage by CCl**_**4**_**and preventive effect of chloroform extract of*Launaea procumbens*showing that Lanes (from left) DNA marker (M), control (1-4), CCl**_**4**_**(5, 8), 200 mg/kg b.w., LPCE + CCl**_**4**_**(9, 10).**

The effects of LPCE and silymarin against CCl_4_-induced toxicities on qualitative DNA damage are shown in Figure 3 whereas those on quantitative damage are provided in Table 3. DNA damage was not observed in the control group. However, the CCl_4_ group showed extensive DNA damage which was significantly (*P* < 0.01) reduced by LPCE depending on the dose as shown by a band pattern and quantification of different groups when compared with the CCl_4_ group.

### Effect of LPCE on hepatic histoarchitecture

**Figure 4:**
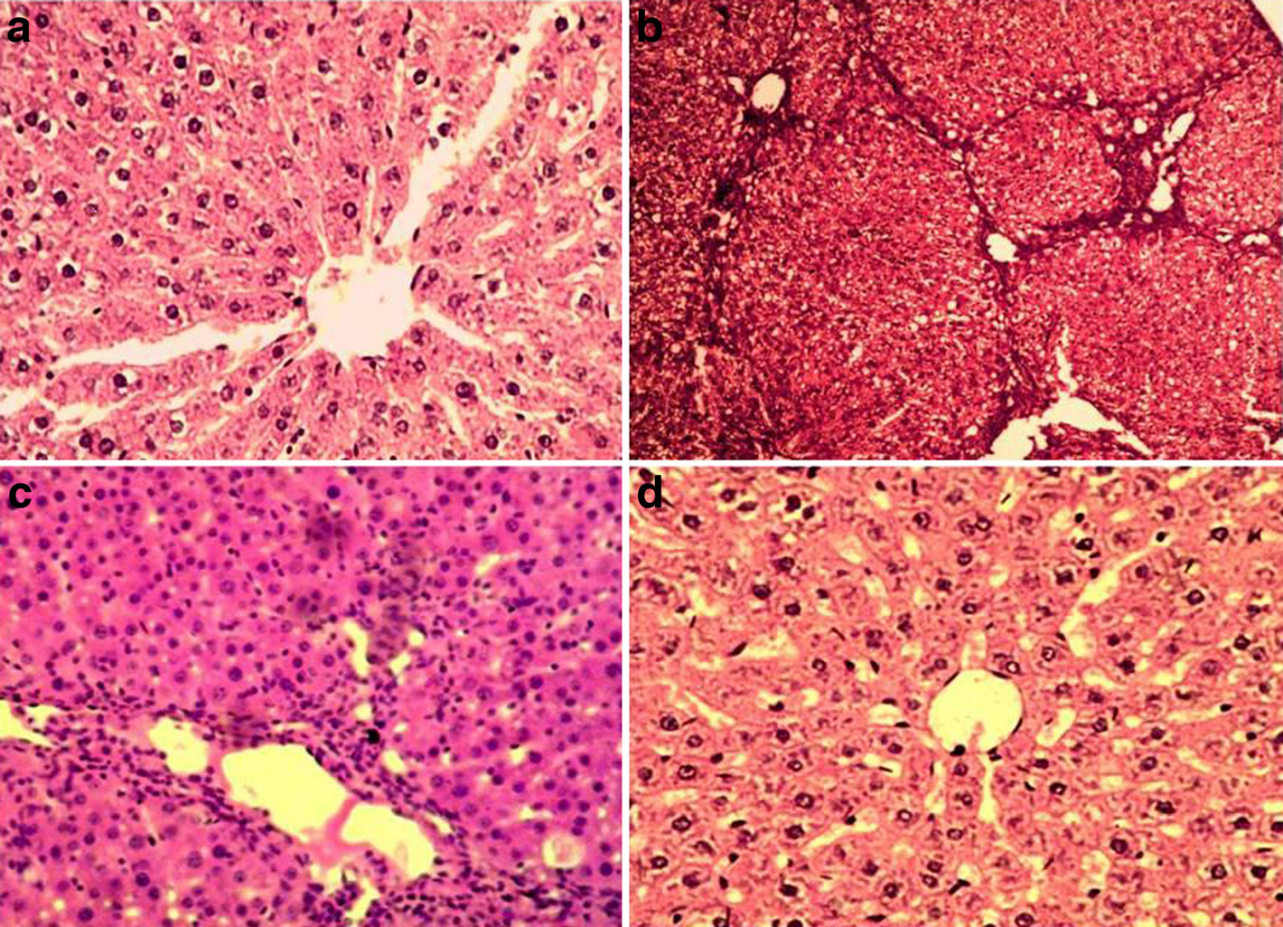
**Histopathological changes caused by CCl**_**4**_**and preventive effect of chloroform extract of*Launaea procumbens*in different groups.** Control **(a),** CCl_4_**(b, c)**, 200 mg/kg b.w., LPCE + CCl_4_**(d).**

Sections of liver stained with H&E were examined for hepatic damage such as necrotic cells, inflammation, neutrophil infiltration, cellular hypertrophy, fibrosis and fatty infiltration (Table 7). Figure 4a depicts the typical normal histology of a liver of untreated control rats. In rats administered CCl_4_, hepatic injury was marked and widespread, and included fatty changes, cellular hypertrophy, necrotic foci, neutrophil infiltration, and fibrosis (Figures 4b and c). Conversely, LPCE and silymarin treatment in combination with CCl_4_ to rats attenuated the hepatic injuries with considerably less or no fatty changes or dilation of blood vessels as well as uniform morphology of hepatocytes similar to those seen in the control group (Figure 4d).

**Table 7:** Effect of LPCE on histology of liver

**Treatment**	**Fatty changes**	**Cellular hypertrophy and necrosis**	**Blood vessel congestion**	**Degeneration of lobules**	**Fibrosis**	**Inflammatory cell infiltrations**
Control	-	-	-	-	-	-
3 ml/kg CCl_4_	+++	+++	+++	+++	+++	+++
100 mg/kg Silymarin+ CCl_4_	+	-	-	-	-	-
100 mg/kg LPCE+CCl_4_	-/+	-	-	-	-	-
200 mg/kgLPCE+CCl_4_	-/+	-	-	-	-/+	-
200 mg/k g LPCE alone	-	-	-	-	-	-

**-,** normal; +/-, mild; ++, medium; +++, severe disruption.

## Discussion

Dietary polyphenols and phenolic compounds are considered to have health-promoting effects in humans. The biological properties of these plant constituents are dependent upon their absorption in the intestine [29,30] and have been reviewed recently [31]. The HPLC data of the present study revealed that LPCE possesses five important polyphenolic compounds (myricetin, kaempherol, rutin, hyperoside, catechin) which have a unique role in detoxification [32]. Studies have revealed the presence of the same phytochemicals during HPLC characterization of medicinal plants [33,34]. Studies have also indicated that flavonoids are potent antioxidant agents [35]. Hence, natural antioxidants such as polyphenols are often added to foods to stabilize them, and there is considerable interest in their potential role as functional foods or “nutraceuticals” [36].

Analyses of liver damage in the present study revealed that CCl_4_ is a potent hepatotoxin and is used extensively for the characterization of hepatoprotective drugs [37]. Poisoning by CCl_4_ causes multisystem disorders involving the liver, kidneys, brain, lungs, adrenal glands, and myocardium [38]. Single exposure of CCl_4_ can rapidly lead to severe centrizonal necrosis and steatosis, and affects the activity of biochemical enzymes, causes breakage of DNA strands, and increases telomerase activity [39]. The results of the present study revealed that CCl_4_ administration caused hepatic injury which caused an elevation of levels of serum marker enzymes such as ALT, AST, ALP, γ-GT and LDH. CCl_4_ administration was shown to cause severe acute liver damage in rats as demonstrated by significant elevation of serum levels of AST and ALT [40,41]. These enzymes were significantly restored by treatment with LPCE, thereby revealing its hepatoprotective ability. High levels of LDL-C and a lower concentration of HDL-C were strongly associated with hepatotoxicity and cardiovascular disease because these biomolecules promote atheroma development in arteries. Presence of significantly higher serum concentrations of LDH, TGs, TC and LDL and decreased levels of HDL-C upon co-treatment with various doses of LPCE demonstrated the hepatoprotective effect of LP*.* Similar results were reported by Sreelatha et al. [42] while working on the hepatoprotective effects of bioactive compounds of plants against CCl_4_-induced hepatic injury in rats.

SOD, catalase and GSH-Px constitute a “mutually supportive team” of antioxidant defense against ROS [43]. Administration of CCl_4_ into rat livers increased lipid peroxidation, resulting in accumulation of superoxide radicals and consequently decreased their activities in the liver [44]. Our data revealed that CCl_4_ treatment significantly decreased the activities of catalase, SOD, GSH-Px, GSR and QR in liver tissues. Co-administration of various doses of LPCE markedly decreased the toxicity of CCl_4_ and enzymatic activities. The ameliorating effects of different plant metabolites on these enzymes against the toxicity of CCl_4_ have also been documented [45]. It has been accepted that the injuries induced by CCl_4_ are attributed to its conversion into the highly toxic CCl_3_^·^ and ^·^OOCCl_3_ by the phase-I cytochrome P450 system in tissues. These free radicals can bind with PUFAs to produce alkoxy (R^·^) and peroxy (ROO^·^) radicals that, in turn, generate lipid peroxide and hydroperoxide, which cause damage to cell membranes and various liver diseases [46]. Elevation of TBARS the end and main oxidative degradation product of lipid peroxidation, functions as a marker of oxidative injury of cellular membranes resulting during the peroxidation of PUFA while a reduction of GSH is an important protein thiol is an important indicator of oxidative stress [47]. GSH acts as a non-enzymatic antioxidant that reduces the toxicity due to H_2_O_2_, hydroperoxides (ROOH) and xenobiotics [48,49]. The data of the present study showed that CCl_4_ significantly decreased GSH contents and increased TBARS contents as compared with the control. Administration of LPCE significantly increased GSH contents and reduced TBARS contents, and this could be due to the presence of various flavonoids in LPCE. Similar results have been reported after the co-administration of propolis against oxidative stress caused by CCl_4_[50]. Lipid peroxidation induced by the free radicals of CCl_4_ combine with DNA to form adducts [17,51]. Our results showed that the DNA of rats in the CCl_4_ group had more damage (as assessed by quantitative means) as compared with the control group. Administration of LPCE significantly reduced the % DNA fragmentation as revealed by the banding pattern of the DNA ladder assay. Similar results have been reported by Khan et al. [39] while studying the protective effects of *Digera muricata* against CCl_4_-induced nephrotoxicity in rats. Histopathological studies revealed that CCl_4_ induces: extensive fatty changes; congestion in blood vessels; cellular hypertrophy; necrotic foci; destruction of lobular architecture; fibrosis; and nuclear degeneration in certain areas. These were markedly diminished by administration of LPCE*.* These data are in good agreement with the activities of serum aminotransferases and hepatic lipid peroxidation levels. Other authors have revealed findings which are in agreement with our findings [52] while evaluating the protective effect of medicinal plants against CCl_4_-induced hepatotoxicity in rats.

## Conclusion

The present study revealed that LPCE recovered enzyme activities in the liver, improved DNA fragmentation, and improved cellular injuries. This study provided evidence in favor of the pharmacological use of LPCE as herbal medicine in the treatment of liver disorders. The presence of antioxidant compounds (catechin, kaempferol, rutin, hyperoside, myricetin) may be responsible for the effectiveness of LPCE against liver disorders.

## Competing interests

The authors declare that they have no competing interests.

## Authors’ contributions

RAK made a significant contribution to acquisition of and analyses of data and drafting of the manuscript. MRK made a substantial contribution to the conception and design of the study, interpretation of data, as well as drafting and revising of the manuscript. SS participated in the study design as well as the collection and analyses of data. All authors read and approved the final manuscript.

## Pre-publication history

The pre-publication history for this paper can be accessed here:

http://www.biomedcentral.com/1472-6882/12/114/prepub
